# Substrate-Dependent Evolution of Cytochrome P450: Rapid Turnover of the Detoxification-Type and Conservation of the Biosynthesis-Type

**DOI:** 10.1371/journal.pone.0100059

**Published:** 2014-06-30

**Authors:** Ayaka Kawashima, Yoko Satta

**Affiliations:** Department of Evolutionary Studies of Biosystems, The Graduate University for Advanced Studies (Sokendai), Shonan Village, Hayama, Kanagawa, Japan; University of South Florida College of Medicine, United States of America

## Abstract

Members of the cytochrome P450 family are important metabolic enzymes that are present in all metazoans. Genes encoding cytochrome P450s form a multi-gene family, and the number of genes varies widely among species. The enzymes are classified as either biosynthesis- or detoxification-type, depending on their substrates, but their origin and evolution have not been fully understood. In order to elucidate the birth and death process of cytochrome *P450* genes, we performed a phylogenetic analysis of 710 sequences from 14 vertebrate genomes and 543 sequences from 6 invertebrate genomes. Our results showed that vertebrate detoxification-type genes have independently emerged three times from biosynthesis-type genes and that invertebrate detoxification-type genes differ from vertebrates in their origins. Biosynthetic-type genes exhibit more conserved evolutionary processes than do detoxification-type genes, with regard to the rate of gene duplication, pseudogenization, and amino acid substitution. The differences in the evolutionary mode between biosynthesis- and detoxification-type genes may reflect differences in their respective substrates. The phylogenetic tree also revealed 11 clans comprising an upper category to families in the cytochrome P450 nomenclature. Here, we report novel clan-specific amino acids that may be used for the qualitative definition of clans.

## Introduction

Enzymes in the cytochrome P450 (CYP) family are heme-binding proteins. The first CYP protein was discovered in rat liver microsomes [1], and it was later functionally characterized as a monooxygenase [2]. Monooxygenases incorporate one of the two atoms of molecular oxygen into the substrate, which results in hydroxylation in the most cases. *CYP* genes form a multi-gene family and encode proteins with amino-acid sequence identities higher than 40%. Each family comprises subfamilies with amino-acid sequence identities higher than 55%. In the classification of CYPs, a clan is defined as a higher-order category of CYP families [3]. Although clans can be useful for defining the relationships among *CYP* genes in different phyla within each kingdom [4], the definition of “clan” is rather arbitrary compared with the definitions of “family” and “subfamily.”

*CYP* genes are present in vertebrates, invertebrates, plants, fungi, and even some prokaryotes [5]. The number of known *CYP* genes in metazoan, plant, and fungus genomes is moderately large. For example, there are 115 *CYP* genes in the human genome, 97 in the sea squirt (*Ciona intestinalis*) (Cytochrome P450 Homepage: http://drnelson.uthsc.edu/cytochromep450.html [6]), 120 in the sea urchin (*Strongylocentrotus purpuratus*) [7], 457 in rice (*Oryza sativa*) [6], 272 in *Arabidopsis thaliana* [8], and 159 in *Aspergillus oryzae* [9]. In contrast, there are relatively a small number of *CYP* genes in eubacteria or Archaea, ranging from none in *Escherichia coli* to 33 in *Streptomyces avermitilis* [9]. Among metazoan *CYP* genes, *CYP51* is particularly conserved and participates in the synthesis of cholesterol, which is an essential component of the eukaryotic cell membrane. A possible prokaryotic homolog (*CYP51B1*) to the metazoan *CYP51* is reported in the genome of *Mycobacterium tuberculosis* [10]. It is therefore thought that *CYP51* is the most ancient *CYP* gene. Although the functional role of CYPs in prokaryotes is not well defined [11], [12], [13], the presence of eukaryotic *CYP* genes in prokaryotes indicates that the emergence of *CYP*s preceded the origin of eukaryotes [14]. However, it has also been suggested that bacterial *CYP51* arose through lateral transfer from plants [15]. Indeed, the absence of *CYP* genes in some bacteria, such as *E. coli*, suggests that they are not essential in prokaryotes.

CYPs are classified into two types, the detoxification type (D-type) and the biosynthesis type (B-type), on the basis of their substrates [4], [16], [17]. In humans, the D-type detoxifies xenobiotics such as plant alkaloids, aromatic compounds, fatty acids, and especially drugs. On the other hand, the B-type is involved in the biosynthesis of physiologically active chemicals such as steroids, cholesterols, vitamin D3, and bile acids. In general, however, only a few CYPs have well-defined substrates, and even in humans, the substrates of some CYPs remain unidentified. For this reason, the nomenclature of vertebrate CYPs (family or subfamily) is largely determined by significant phylogenetic clustering with known functional sequences in humans. We also adopt this phylogenetic method to classify each vertebrate gene as either B- or D-type.

Fission yeasts have two B-type *CYP* genes (*CYP51F1* and *CYP61A3*) but no D-type genes. D-type genes are therefore thought to have emerged from a B-type gene in eukaryotes [4], [14], [16]. Nelson *et al.* [9] performed a phylogenetic analysis of 1,572 metazoan *CYP*s and reported the presence of 11 clans. They further investigated the origin and evolutionary processes of these clans. However, the length of intermodal branches connecting the different clans were too short to unambiguously discern the phylogenetic relationships between clans, making it difficult to fully characterize early *CYP* evolution.

In vertebrates, the number of *CYP* genes per genome varies greatly between species; for example, humans have 115, mice have 185, and zebrafish have 81 (Cytochrome P450 Homepage). Among the 115 human *CYP*s, 57 are functional and 58 are pseudogenes, suggesting rapid gene turnover. The 115 genes constitute 18 families, and the number of subfamilies within each family ranges from 1 to 13. These *CYP* genes are distributed on all chromosomes except chromosomes 5, 16, and 17. Five clusters of closely related genes are located on chromosomes 1, 7, and 10 (one cluster each) and chromosome 19 (two clusters) [18], [19]. One such cluster on chromosome 19 has been studied in primates and rodents as well from an evolutionary viewpoint, indicating that an initial tandem duplication occurred in an early mammalian ancestor and that gene duplications and/or rearrangements frequently occurred in a lineage-specific manner [20].

In this paper, we aim to elucidate the birth and death processes of vertebrate *CYP* genes. In particular, we compare and contrast the origin and evolution of B- and D-types, and present an evolutionary model of vertebrate *CYP* genes.

## Materials and Methods

### Sequence datasets and identification of B- and D-type genes in vertebrates and invertebrates

The nucleotide sequences of 115 *CYP* genes in the human genome were obtained from the Cytochrome P450 Homepage. Using these sequences as queries, we performed a basic local alignment search tool (BLAST) search by using BLASTn and downloaded coding sequences (CDS) of homologous nucleotide sequences from 14 vertebrate species (*Pan troglodytes*: CHIMP2.1.4, *Macaca mulatta*: MMUL_1, *Callithrix jacchus*: C_jacchus3.2.1, *Bos taurus*: UMD 3.1, *Canis lupus familiaris*: CanFam3.1, *Mus musculus*: GRCm38.p2, *Rattus norvegicus*: Rnor_5.0, *Monodelphis domestica*: BROADO5, *Gallus gallus*: Galgal4, *Taeniopygia guttata*: 3.2.4, *Anolis carolinensis*: AnoCar2.0, *Xenopus tropicalis*: JGI 4.2, *Oryzias latipes*: MEDAKA1.70, and *Danio rerio*: Zv9) from NCBI (http://www.ncbi.nlm.nih.gov/) or ENSEMBL databases (http://www.ensembl.org/index.html). In the BLAST search, the top two hits and the top five hits were retrieved when B- and D-type genes were used as queries, respectively. The nucleotide sequences of ref-seq from NCBI were obtained, and sequences from ENSEMBL were filtered by length (>1000 bp) and their identity with human genes. The extent of sequence identity was dependent on the divergence time between each vertebrate species and humans. For example, in fish, we filtered out sequences with identity >60%. Orthology was confirmed by the presence of a syntenic region and the presence of adjacent loci, if any.

The following invertebrate species were included in the analysis: amphioxus (*Branchiostoma floridae*), sea squirt (*C. intestinalis*), sea urchin (*S. purpuratus*), sea anemone (*Nematostella vectensis*), water flea (*Daphnia pulex*), and fruit fly (*Drosophila melanogaster*). Protein sequences obtained from the Cytochrome P450 Homepage were used for the analysis of invertebrate CYPs. Only protein sequences >350 amino acids in length were included in the phylogenetic analysis. Because of the too extensive sequence divergence between vertebrate and invertebrate *CYP* genes, BLAST searches of the NCBI and ENSEMBL databases were not performed.

### Molecular evolutionary analysis

Vertebrate nucleotide sequences and invertebrate amino acid sequences in *CYP* coding regions were aligned separately using ClustalW [21] implemented in MEGA5 [22], and each alignment was further edited by hand. In the alignment of the vertebrate nucleotide sequences, we first translated them into the amino acid sequences and after checked by eye, reconverted them to the nucleotide sequences. We excluded sites at which >20% of the operating taxonomic units (OTUs) showed gaps. As a result, 28.7% of the aligned sites showed >60% identity, 48.5% showed >50% identity, and 71.9% showed >30% identity (data not shown). We then constructed Neighbor-joining (NJ) trees [23] using either nucleotide differences per site (p-distance) [24] or amino acid distances (JTT distance) [25]. We performed missing data treatment under both the pairwise deletion and complete deletion options. The maximum likelihood (ML) [26] method was used to test the tree topology. All methods for tree construction were implemented in MEGA5 [22].

### Pseudogenization or deletion of genes

The nucleotide sequences of the *CYP* pseudogenes in the human genome were obtained from the Cytochrome P450 Homepage. We selected genes containing >1000 bp out of the 1500 bp CDS. We retrieved orthologous genes from other vertebrate genomes by performing BLAST searches, using the human sequences as queries. The orthologous sequences were aligned with their human counterparts by ClustalW. Based on this alignment, we searched other vertebrates for nonsense or frame-shift mutations found in humans. To estimate the time of pseudogenization, we calculated the ratio of non-synonymous substitutions to synonymous substitutions, always per site, for pairs comprising a pseudogene and an orthologous functional gene. Using this ratio, we estimated the pseudogenization time for all *CYP* pseudogenes from the formula in Sawai *et al.* [27]. We used the TimeTree (http://www.timetree.org/index.php [28]) as a reference for calibrating species divergence time. When an orthologous gene is absent (deletion) in any non-human vertebrate, we searched for the syntenic region in the genome in order to confirm the deletion.

### Estimation of functional constraint

In order to compare the amino-acid substitution rate for each functional *CYP* gene in primates, we normalized the rate with the synonymous substitution rate. This normalization for each gene measured the degree of “functional constraint”. To be complete, we assumed that the gene tree is the same as the species tree for four primates (humans, chimpanzees, rhesus macaques, and marmosets) and placed the numbers of synonymous and non-synonymous substitutions on each branch by the least squares method [29]. The degree of functional constraint 1 - *f* is obtained from the ratio (*f*) of the sum of non-synonymous substitutions to that of synonymous substitutions of all branches in each tree. Finally, we compared the degree of functional constraint or directly the *f* value between B-type and D-type genes by using the Mann–Whitney *U* test [30].

## Results

### Vertebrate D-type genes emerged independently three times from B-type genes

Among the 57 functional *CYP* genes in the human genome, 35 are D-type genes and 22 are B-type genes. This classification is based on the description of the enzyme substrate [31], if any, and subfamily or family classification [6]. D-type genes constitute four *CYP* families: *CYP1* (3 genes), *CYP2* (16 genes), *CYP3* (4 genes), and *CYP4* (12 genes). B-type genes are grouped into 14 families: *CYP*5 (1 gene), *CYP7* (2 genes), *CYP8* (2 genes), *CYP11* (3 genes), *CYP17* (1 gene), *CYP19* (1 gene), *CYP20* (1 gene), *CYP21* (1 gene), *CYP24* (1 gene), *CYP26* (3 genes), *CYP27* (3 genes), *CYP39* (1 gene), *CYP46* (1 gene), and *CYP51* (1 gene) (Table S1). Using the definition proposed by Nelson [32], the 57 CYPs can be classified into 10 clans: clans 2, 3, 4, mito, 7, 19, 20, 26, 46, and 51. Clan “mito” contains genes encoding enzymes that operate in mitochondria. Of the 10 clans, 6 (2, 3, 4, mito, 7, and 26) contain more than two families, whereas 4 (19, 20, 46, and 51) contain only one single family. The amino acid alignment of the 57 functional *CYP* genes showed that four amino aid sites are conserved. Two of these (310F and 316C) sare located near the heme-binding region (Figure S1). The latter site (316C) is known to be structurally close to the iron ion in the heme-binding region and to operate as an active center of the enzyme [33]. This conserved cysteine is said as the proximal Cys [33]. The other two sites (242E and 245R) are located about 80 amino acids upstream from the proximal Cys. Although it is unknown whether these amino acids are involved in any specific function, their conservation suggests some evolutionary or functional importance. Furthermore, several clan-specific amino acids were found in the 57 functional human *CYP*s (Figure S2). Some of them were conserved not only in vertebrates but also in metazoans, although the number of conserved sites correlates with the number of genes in each clan (data not shown).

**Figure 1:**
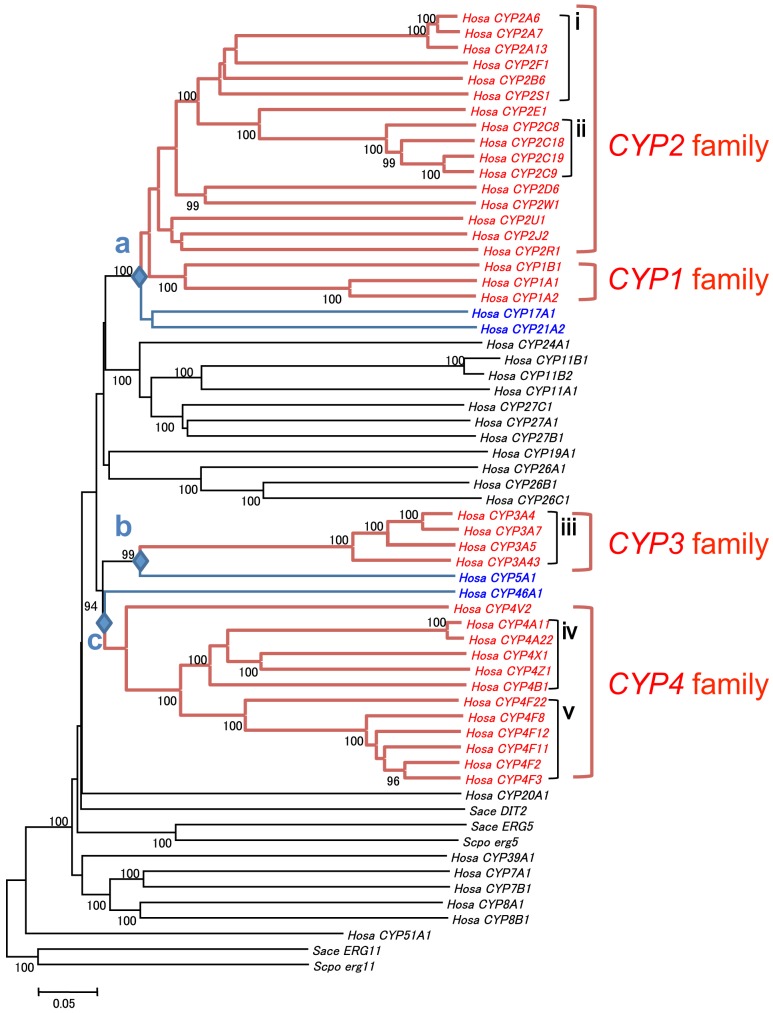
Phylogenetic tree of *cytochrome P450* genes in humans. The tree includes all functional *CYP* genes in humans (*Hosa*) and all yeasts (*Sace*, *Saccharomyces cerevisiae*; *Scpo*, *Schizosaccharomyces pombe*). The tree was constructed using the NJ method for nucleotide differences between the CDS and rooted with yeast *CYP51* gene sequences (*Sace ERG11* and *Scpo erg11*). Red text indicates D-type *CYP* genes, and black and blue text indicate B-type *CYP* genes. The *CYP1*–*4* families are indicated by a red bracket on the right side of the tree. Three diamonds (*a*, *b*, and *c*) indicate duplications of B- and D-type genes. The B-type genes that were the ancestors of D-type genes are indicated with a blue line and character. Black brackets and roman numerals (i–v) at the tips of the tree show five clusters of D-type genes: i, the *CYP2* family on chromosome 19q; ii, the *CYP2C* subfamily on chromosome 10q; iii, the *CYP3A* subfamily on chromosome 7q; iv, the *CYP4* family on chromosome 1p; v, the *CYP4F* subfamily on chromosome 19p. The number near each node indicates the bootstrap value (>94%) supporting the node.

To characterize the phylogenetic relationships among the 57 functional *CYP* genes in the human genome, an NJ tree was constructed based on the total nucleotide differences (p-distances) between the CDSs (Figure 1). In the resulting tree, members of each family formed monophyletic groups with respect to other families, and each monophyletic group was supported by a relatively high bootstrap value. The phylogeny showed that D-type genes emerged independently from B-type genes at least three times: first, an ancestral gene of *CYP17A1* and *CYP21A1* was duplicated, generating the ancestor of the *CYP1* and *CYP2* families (node *a* in the tree, Figure 1). Second, the *CYP3A* subfamily arose from the common ancestor of *CYP3* and *CYP5* (node *b* in the tree). Third, an ancestor of *CYP46A1* was duplicated, generating the ancestor of the *CYP4* family (node *c* in the tree). All nodes (*a*, *b*, and *c*) were supported by high bootstrap values (94∼100% in Figure 1). In addition to these bootstrap values, amino acids that could distinguish B- from D-type genes were also identified (Figure S3). For example, an amino acid site in the middle of the sequence supported node *a*. In the D-type genes, F was shared by all members of the *CYP1* family whereas V was shared by all members of the *CYP2* family. In contrast, the B-type *CYP*s, *CYP17A1*, and *CYP21A1*, shared T at that site. Similarly, several other amino acid changes that support nodes *a*, *b*, and *c* were observed (Figure S3).

**Figure 2:**
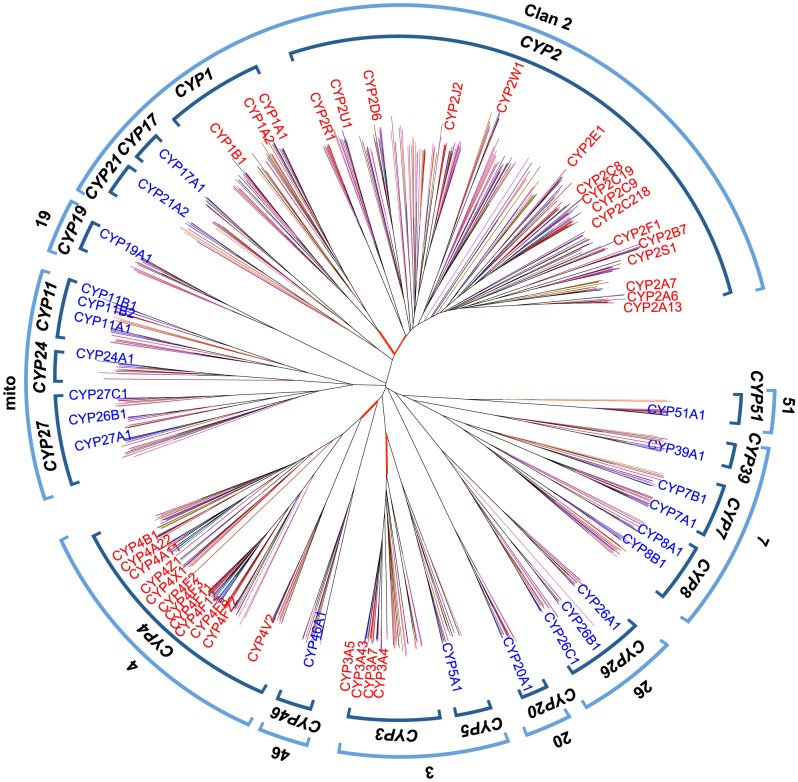
The phylogenetic tree of B- and D-type *CYP* genes in vertebrates. An internal bracket at the tips of the tree indicates the *CYP* family in vertebrates, and an external bracket indicates clusters for a clan. D-type *CYP* genes in humans are shown in red, and B-type *CYP* genes are shown in blue. Red-shaded branches indicate the divergence of D-type from B-type. Text colors indicate the following: red for D- and blue for B-type in humans; dark brown for *Bos taurus*; light blue for *Canis lupus familiaris*; pink for *Mus musculus*; aqua for *Rattus norvegicus*; dark red for *Monodelphis domestica*; dark orange for *Gallus gallus*; purple for *Taeniopygia guttata*; brown for *Anolis carolinensis*; blue-purple for *Xenopus tropicalis*; red-purple for *Oryzias latipes*; orange for *Danio rerio*.

To investigate the duplicaion times of three major D-types from their ancestral B-types, orthologs and paralogs of human B-type and D-type *CYP* genes were retrieved from 14 vertebrate genomes. This resulted in a total of 710 *CYP* nucleotide sequences so that we examined twice as many vertebrate sequences as in the previous study (388) [9]. The presence or absence of vertebrate orthologs to the 57 functional human genes is summarized in Table 1, showing that almost all 14 genomes contain orthologs of B-type genes. We used the pairwise deletion option and constructed a phylogenetic tree (Figure 2); its topology readily confirmed the orthologous relationship between human and other vertebrate B-type genes. However, it was difficult to identify orthologous relationships between D-type genes from humans and other vertebrates, especially in the *2A*, *2C*, *3A*, and *4F* subfamilies, owing to frequent species-specific duplications. Nevertheless, monophyletic relationships within each D-type family (*CYP1*–*4*) were observed with relatively high bootstrap values (>80%), so that vertebrate genes in each monophyletic group are classified as the D-type. The phylogenetic analysis revealed that human D- and B-type genes had already emerged when vertebrates diverged, and that three duplication events occurred in the B-type genes from which the D-type genes were originated.

**Table 1:** Presence or absence of vertebrate orthologs to human *CYP* genes.

*CYP family*		*Species name*													
	*genes*	*Patr*	*Mamu*	*Caja*	*Bota*	*Cafa*	*Mumu*	*Rano*	*Modo*	*Anca*	*Gaga*	*Tagu*	*Xetr*	*Orla*	*Dare*
*1*	*A1*	*1*	*1*	*1*	*1*	*1*	*1*	*1*							
	*A2*	*1*	*1*	*1*	*1*	*1*	*1*	*1*							
	*A1 or A2*								*2*	*3*	*1*	*1*	*1*	*1*	*1*
	*B1*	*1*	*1*	*1*	*1*	*1*	*1*	*1*	*1*	*1*	*0*	*1*	*1*	*1*	*1*
	*Others*	*0*	*0*	*0*	*1*	*0*	*0*	*0*	*1*	*2*	*1*	*0*	*3*	*2*	*3*
*2*	*A6*	*0*	*1*	*0*	*0*	*0*									
	*A7*	*1*	*2*	*0*	*0*	*1*									
	*A13*	*1*	*2*	*1*	*1*	*1*									
	Other A*						*4*	*2*	*1*	*0*	*0*	*0*	*0*	*0*	*0*
	*B6*	*1*	*1*	*1*	*1*	*1*	*4*	*4*	*2*	*0*	*0*	*0*	*0*	*0*	*0*
	*C8*	*1*	*1*	*1*											
	*C9*	*1*	*1*	*1*											
	*C18*	*1*	*1*	*1*											
	*C19*	*1*	*1*	*1*											
	Other C*				*7*	*2*	*9*	*6*	*8*	*0*	*0*	*0*	*0*	*0*	*0*
	*D6*	*1*	*1*	*1*	*2*	*0*	*5*	*5*	*1*	*1*	*1*	*1*	*5*	*0*	*0*
	*E1*	*1*	*1*	*1*	*1*	*1*	*1*	*1*	*1*	*0*	*0*	*0*	*0*	*0*	*0*
*2*	*F1*	*1*	*1*	*1*	*1*	*3*	*1*	*1*	*1*	*1*	*0*	*0*	*0*	*0*	*0*
	*J2*	*1*	*1*	*1*	*5*	*1*	*6*	*3*	*6*	*1*	*4*	*2*	*1*	*0*	*0*
	*R1*	*1*	*1*	*1*	*1*	*1*	*1*	*0*	*1*	*1*	*1*	*1*	*1*	*1*	*1*
	*S1*	*1*	*1*	*1*	*1*	*1*	*2*	*1*	*1*	*0*	*0*	*0*	*0*	*0*	*0*
	*U1*	*1*	*1*	*1*	*1*	*1*	*1*	*1*	*1*	*0*	*0*	*1*	*1*	*1*	*1*
	*W1*	*1*	*1*	*1*	*1*	*1*	*1*	*1*	*1*	*2*	*1*	*1*	*0*	*0*	*0*
	*Others*	*1*	*1*	*1*	*1*	*2*	*1*	*2*	*1*	*15*	*7*	*7*	*18*	*10*	*20*
*3*	*A4*	*1*	*1*	*1*											
	*A5*	*1*	*1*	*1*											
	*A7*	*1*	*1*	*1*											
	*A43*	*1*	*1*	*1*											
	*Other A* *				*3*	*4*	*8*	*4*	*4*	*3*	*2*	*1*	*5*	*1*	*1*
	*Others*	*0*	*0*	*0*	*0*	*0*	*0*	*0*	*0*	*0*	*0*	*0*	*0*	*2*	*5*
*4*	*A11*	*1*	*1*	*1*	*2*	*1*									
	*A22*	*1*	*1*	*1*	*2*	*1*									
	*A11 or 22*						*5*	*1*	*0*	*0*	*0*	*0*	*0*	*0*	*0*
	*OtherA* *	*0*	*0*	*0*	*0*	*3*									
	*B1*	*1*	*1*	*1*	*1*	*1*	*2*	*1*	*1*	*6*	*2*	*2*	*4*	*2*	*3*
	*F2*	*1*	*1*	*1*											
	*F3*	*1*	*1*	*1*											
	*F8*	*1*	*1*	*1*											
	*F11*	*1*	*1*	*1*											
	*F12*	*1*	*1*	*1*											
	*F22*	*1*	*1*	*1*											
	*Other F* *				*6*	*3*	*9*	*8*	*6*	*1*	*0*	*1*	*3*	*1*	*1*
	*V2*	*1*	*1*	*1*	*1*	*1*	*1*	*1*	*0*	*1*	*1*	*0*	*2*	*1*	*2*
	*X1*	*1*	*1*	*1*	*1*	*2*	*1*	*1*	*2*	*0*	*0*	*0*	*0*	*0*	*0*
	*Z1*	*1*	*1*	*1*	*0*	*0*	*0*	*0*	*0*	*0*	*0*	*0*	*0*	*0*	*0*
	*5A1*	*1*	*1*	*1*	*1*	*1*	*1*	*1*	*1*	*1*	*1*	*1*	*1*	*1*	*1*
*7*	*A1*	*1*	*1*	*1*	*1*	*1*	*1*	*1*	*1*	*1*	*1*	*1*	*1*	*1*	*1*
	*B1*	*1*	*1*	*1*	*1*	*1*	*1*	*1*	*1*	*1*	*1*	*1*	*0*	*0*	*1*
*8*	*A1*	*1*	*1*	*1*	*1*	*1*	*1*	*1*	*1*	*0*	*0*	*0*	*1*	*1*	*1*
	*B1*	*1*	*1*	*1*	*1*	*1*	*1*	*1*	*2*	*1*	*1*	*1*	*2*	*1*	*3*
*11*	*A1*	*1*	*1*	*1*	*1*	*1*	*1*	*1*	*1*	*0*	*1*	*1*	*1*	*1*	*1*
	*B1*	*1*	*1*	*1*	*1*	*0*	*1*	*1*	*1*	*1*	*0*	*0*	*0*	*0*	*1* *
	*B2*	*1*	*1*	*1*	*0*	*1*	*1* *	*1* *	*0*	*0*	*0*	*0*	*0*	*0*	*0*
	*17A1*	*1*	*1*	*1*	*2*	*1*	*1*	*1*	*0*	*1*	*1*	*1*	*1*	*1*	*2*
	*19A1*	*1*	*1*	*1*	*1*	*1*	*1*	*1*	*1*	*1*	*1*	*1*	*1*	*2*	*2*
	*20A1*	*1*	*1*	*1*	*1*	*1*	*1*	*1*	*1*	*1*	*1*	*1*	*1*	*1*	*1*
	*21A2*	*1*	*1*	*1*	*1*	*1*	*1*	*1*	*1*	*1*	*1*	*1*	*1*	*2*	*2*
	*24A1*	*1*	*1*	*1*	*1*	*1*	*1*	*1*	*1*	*1*	*0*	*2*	*2*	*1*	*1*
*26*	*A1*	*1*	*1*	*1*	*1*	*1*	*1*	*1*	*1*	*1*	*1*	*1*	*1*	*1*	*1*
	*B1*	*1*	*1*	*1*	*1*	*1*	*1*	*1*	*0*	*0*	*1*	*1*	*1*	*1*	*1*
	*C1*	*1*	*0*	*1*	*1*	*1*	*1*	*1*	*1*	*1*	*1*	*1*	*1*	*1*	*1*
*27*	*A1*	*1*	*1*	*1*	*1*	*1*	*1*	*1*	*1*	*1*	*1*	*0*	*2*	*1*	*2*
	*B1*	*1*	*1*	*1*	*1*	*1*	*1*	*1*	*1*	*1*	*0*	*0*	*1*	*1*	*1*
	*C1*	*1*	*1*	*1*	*1*	*1*	*0*	*0*	*1*	*1*	*1*	*1*	*1*	*1*	*1*
	*39A1*	*1*	*1*	*1*	*1*	*1*	*1*	*1*	*1*	*1*	*1*	*1*	*1*	*0*	*1*
	*46A1*	*1*	*1*	*1*	*1*	*1*	*1*	*1*	*0*	*1*	*1*	*1*	*2*	*2*	*2*
	*51A1*	*1*	*1*	*1*	*1*	*1*	*1*	*1*	*1*	*1*	*1*	*1*	*1*	*1*	*1*

Others: *CYP* genes are not orthologs to the human *CYP* genes listed here but are subfamily members belonging to each family.

*: Genes are included in the subfamily, but the subfamily number differs from that in humans.

**Figure 3:**
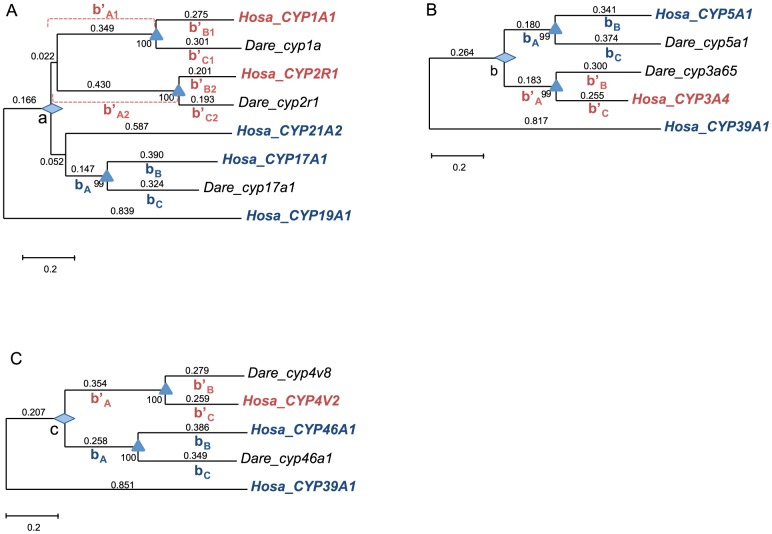
Duplication time of B-type and D-type genes. A) The divergence between *CYP1/2* and *17A1/21A2*. B) The divergence between *CYP3A* and *5A1*. C) The divergence between *CYP4* and *46A1*. The divergence between humans and zebrafish was used as a calibration time ( = 400 mya), and is shown as a triangle in each tree. The duplication event is shown as a diamond. *b*_A_, *b*_B_, and *b*_C_ represent the branch length between the duplication event and species divergence. The branches after species divergence in B-type genes and the branches *b*′_A_, *b*′_B_, and *b*′_C_ represent the length of D-type genes. The number near each branch shows the branch length.

Assuming a molecular clock and that zebrafish and humans diverged 400 million years ago (mya) (TimeTree; http://www.timetree.org/), we calculated the total branch lengths leading to both B- and D-type genes (branch *b*_A_, *b*_B_, and *b*_C_ to B-type and *b*′_A_, *b*′_B_, and *b*′_C_ to D-type in Figure 3A–C) to estimate the timing of the emergence of the *CYP1*–*4* families (nodes *a*, *b*, and *c* in Figure 1). Since *b*_B_, *b*_C_, *b*′_B_, and *b*′_C_ correspond to 400 million years (myr), each ratio of (*b*_A_+*b*_B_) to *b*_B_, (*b*_A_+*b*_C_) to *b*_C_, (*b*′_A_+*b*′_B_) to *b*′_B_, and (*b*′_A_+*b*′_C_) to *b*′_C_ yielded an estimate of the duplication time. The estimates varied from 623–1316 mya for *a*, 601–664 mya for *b*, and 681–926 mya for *c*. To be conservative, we used the youngest estimate for each node: 623±35 mya for *a*, 601±34 mya for *b*, and 681±37 mya for *c*. As anticipated, these estimates preceded the emergence of vertebrates (608 mya, TimeTree) but occurred after the divergence of vertebrates and chordates (774 mya, TimeTree). This finding suggests that invertebrates do not possess orthologs to vertebrate D-type genes, despite the presence of D-type *CYP*s in insects, which function in insecticide resistance and detoxification of plant alkaloids [34].

### Evolutionary relationship between invertebrate and vertebrate *CYP*s

**Figure 4:**
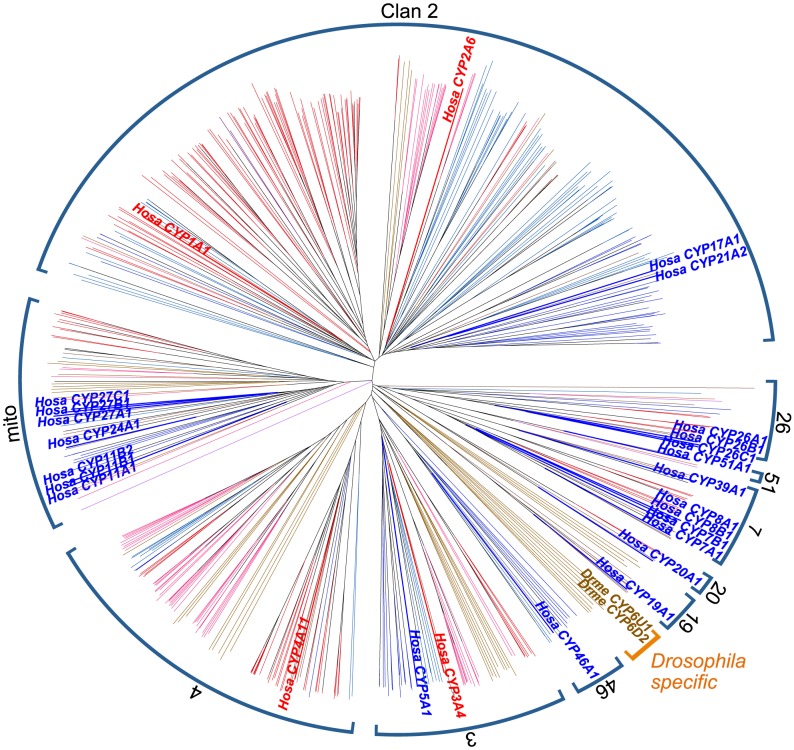
NJ tree of all invertebrate *CYP* genes. D- and B-type *CYP* genes in humans are shown in red and blue text, respectively. The numbers near the brackets indicate clans. Orange character indicates the *Drosophila*-specific clan. Abbreviations and their color (in parentheses) are defined as follows: *Hosa*, *Homo sapien*s (red for D- and blue for B-type); *Brfl*, *Branchiostoma floridae* (dark brown); *Neve*, *Nematostella vectensis* (light blue); *Dapu*, *Daphnia pulex* (pink); *Stpu*, *Strongylocentrotus purpuratus* (aqua); *Ciin*, *Ciona intestinalis* (dark red); *Drme*, *Drosophila melanogaster* (brown); *Sace*, *Saccharomyces cerevisiae*; *Scpo*, *Schizosaccharomyces pombe* (purple). Gene names of *CYP6D2* and *6U1* in *D. melanogaster* are shown in clan 19. Bootstrap values supporting nodes of clusters mentioned in the text are shown.

To further examine the relationships between vertebrate and invertebrate D-type *CYP* genes, we searched for homologs of human D-type *CYP*s in six invertebrate species (amphioxus: *B. floridae*, sea squirt: *C. intestinalis*, sea urchin: *S. purpuratus*, sea anemone: *N. vectensis*, water flea: *D. pulex*, and fruit fly: *D. melanogaster*). A total of 543 *CYP* amino acid sequences were retrieved from the Cytochrome P450 Homepage. A preliminary search to determine the phylogenetic position of vertebrate *CYP*s in the tree that included both vertebrate and invertebrate *CYP*s revealed that each vertebrate *CYP* family formed a monophyletic group. To simplify the phylogenetic analysis, amino acid sequences from these invertebrates were aligned only with sequences from humans, as a representative vertebrate, and the tree was constructed on the basis of amino acid distances (Figure 4).

The amino-acid distance tree shows that 10 clans (clans 2, 3, 4, mito, 7, 19, 20, 26, 46, and 51) are common to vertebrates; the tree also reveals one *Drosophila*-specific clan. A previous study of 1,572 *CYP* sequences also identified 11 clans in metazoans, but with inclusion of clan 74, which was present only in lancelets, sea anemones, and Trichoplax, but absent in vertebrates [9]. In the present analysis, despite the inclusion of both lancelet and sea anemone, clan 74 was not detected. However, a further phylogenetic analysis that included only yeasts, humans, lancelets, and sea anemones identified clan 74, although it was supported by a relatively low bootstrap value (55%). In addition, the genes that comprised the *Drosophila*-specific clan (*CYP6D2*, *6U1*, *28A5*, *28C1*, *28D1*, *308A1*, *309A1*, *350A1*, and *317A1*) were all included in clan 3 [9]. This holds true when we draw trees with different methods (maximum likelihood), although the bootstrap value for this clan is too low (<20%) to confirm this inclusion. We also observed some other differences from the previous study [9]: clan 51 did not include any sea-urchin gene, and clan 20 included neither sea urchin nor sea-anemone gene (Figure 4). The absence of a sea-urchin *CYP51* ortholog can be explained by the incompleteness of the database used here. In fact, a blast search of the NCBI database using human *CYP51* as a query identified a *CYP51* gene (Accession number: NM_001001906) in the recently published sea urchin genome. However, clan 20-like genes were absent from the sea urchin and the sea-anemone genomes in the database. In addition, clan 19 in the present tree appeared to include the *Drosophila* genes (*313A1*, *313B1*, *316A1*, and *318A1*) that were included in clan 4 in the previous study. In fact, the *Drosophila*-specific genes in clan 19 shared 16 of 433 amino acids with human *CYP19* (Figure S4), and these 16 amino acids were conserved among vertebrate CYPs. However, an ML tree supported the presence of the *Drosophila* sequences in clan 4, with very low bootstrap support (6%).

Clans including invertebrate *CYP* genes were supported by low bootstrap values, and clan definitions were dependent on the methods used for tree construction. Thus, the notion of clan becomes ambiguous and ill-defined for distantly related metazoan *CYP* genes.

### The origin of D-type genes in vertebrates and invertebrates

Clan 2 included human *CYP17A1* and *CYP21A2* (B-type) as well as members of the *CYP1* and *CYP2* families (D-type). Similarly, clan 3 included both types of *CYP* genes: *CYP5A1* (B-type) and *CYP3A* subfamilies (D-type). These two cases indicate that the emergence of D-type from B-type genes occurred after the emergence of the clan. However, clan 4 included only the *CYP4* family from humans but not *CYP46A1*, an ancestor of the *CYP4* family. This is the only case where the emergence of the D-type predates clan emergence. In addition, clan 4 included both vertebrate and invertebrate genes. Vertebrate *CYP4* likely acquired its detoxification function in the stem lineage of vertebrates when invertebrate sequences were B-type; alternatively, the ancestor of clan 4 may have already possessed D-type functions when invertebrate genes in clan 4 encoded D-type enzymes.

Fruit flies are known to possess two D-type *CYP* genes, *CYP6D2* and *CYP6U1*, which function in insecticide metabolism. In the tree generated in this study, these *CYP* genes were distantly related to human D-type genes, suggesting that D-type genes in fruit flies emerged independently from those in vertebrates.

### Gene duplications and losses in the B- and D-type lineages during vertebrate evolution

**Figure 5:**
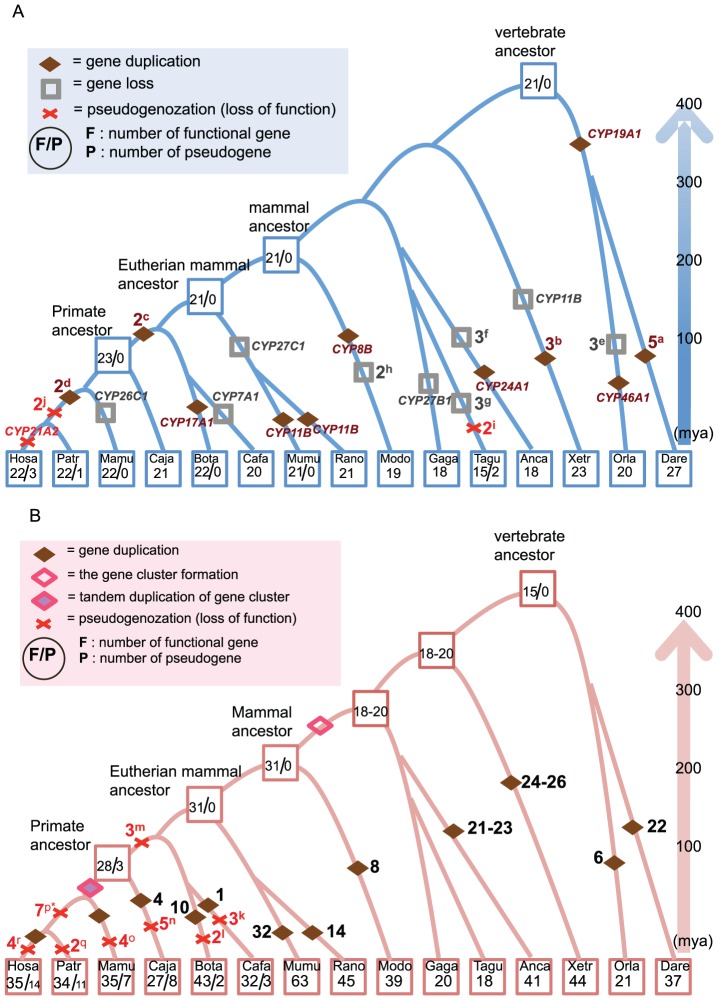
The birth and death processes of *CYP* genes in vertebrates. A) B-type *CYP* genes and B) D-type *CYP* genes. In both figures, numbers inside squares represent the number of functional genes and pseudogenes in each species and its ancestors. Diamonds, crosses, and rectangles indicate gene duplication, pseudogenization, and deletion events, respectively. The number adjacent to each symbol represents the number of events. The letter adjacent to the number indicates the list of *CYP* genes, as follows. a: *CYP8B2*, *CYP8B3*, *CYP17A1*, *CYP27A1*, and *CYP46A1*, b: *CYP8B*, *CYP27A1*, and *CYP46A1*, c: *CYP11B* and *CYP21A2*, d: *CYP51* (two genes), e: *CYP7B*, *CYP11B*, and *CYP39A1*, f: *CYP11A*, *CYP21A2*, and *CYP26*, g: *CYP11B*, *CYP21A2*, and *CYP27* (A or B), h: *CYP17A1* and *CYP26B1*, i: *CYP24A1* and *CYP27A1*, j: *CYP21A1P* and *CYP51* (two genes), k: *CYP4F9P*, *CYP4F23P*, and *CYP4F24P*, l: *CYP4A11* and *CYP2F1P*, m: *CYP2T2P*, *3P*, and *CYP2G1P*, n: *CYP4A11*, *CYP4B1*, *CYP4F22*-like (two genes), and *CYP4F23P*, o: *CYP2A7P1*, *CYP2A13*, *CYP2B6P*, and *CYP4F11*, p: *CYP2B7P1*, *CYP2D8P1*, *CYP2F1P*, *CYP4F9P*, *CYP4F23P*, and *CYP4F24P*, *CYP1D1P were found in *Pan paniscus*, but were absent from *Pan troglodytes*, q: *CYP2B6* and *CYP2C18*, r: *CYP2A7P1*, *CYP2G2P*, and *CYP4Z2P*.

Nearly all of the 14 vertebrate genomes examined here contained 21 orthologs to the 22 functional human B-type genes. On the basis of the presence or absence of *CYP* genes in each vertebrate genome, we parsimoniously estimated the number of genes in each ancestor of amniotes, mammals, eutherian mammals, primates, catarrhini, and hominoids, as well as the number of gains of genes in each taxonomic lineage. The number of ancestral genes remained stable throughout the evolution of vertebrates: the number of genes in each vertebrate ancestor did not change over the course of evolution until the emergence of a primate ancestor. A gene-duplication event occurred in the primate ancestor, generating *CYP11B2*. In the ancestor of hominoids, emergence of new genes occurred twice, generating the ancestors of the *CYP51P1* and *CYP51P2* genes (Figure 5A).

In contrast to the rather stable mode of evolution observed in the stem, lineage-specific gains and losses of genes occurred relatively frequently. For instance, a shared duplication of *CYP19A1* occurred in the lineage leading to the common ancestor of zebrafish and medaka. In addition, lineage-specific gene duplications occurred in the zebrafish (*CYP8B2*, *CYP8B*3, *CYP17A1*, *CYP27A1*, and *CYP46A1*), medaka (*CYP46A1*), frog (*CYP8B1*, *CYP27A1*, and *CYP46A1*), green anole (*CYP24A1*), and opossum (*CYP8B1*) lineages. Interestingly, gene duplications of *CYP8B*, *CYP46A1*, and *CYP27A1* occurred independently several times in a species-specific manner. Similarly, lineage-specific gene losses (deletions) were observed; for instance, deletions occurred in a lineage leading to the medaka (*CYP7B1*, *CYP11B1*, and *CYP39A1*), frog (*CYP11B1*), green anole (*CYP11A1*, *CYP21A2*, *CYP26A1*), chicken (*CYP27B1*), zebra finch (*CYP11B1*, *CYP21A2* and *CYP27*), and opossum (*CYP17A1* and *CYP26B1*). Several deletions affecting the same genes (*CYP11B1* and *CYP21A2*) occurred independently in medaka, frog, green anole, and zebrafinch.

Although only a limited number of genomic sequences are available, we identified 19 gene gains and 16 losses among the 15 available genomes, including the human genome. Assuming that the total branch length in the vertebrate tree is 2,685 myr (for individual species divergence times, see Figure S5), we estimated the rate of gene gains and losses to be 0.7 and 0.6 per 100 myr, respectively.

Using a similar analysis, we also examined the 14 vertebrate genomes for the presence of paralogs and orthologs of 35 human D-type genes. This analysis revealed that the number of genes varies from 15 to 31 in ancestral species, and from 18 to 63 in extant species (Figure 5B). In contrast to the relatively stable evolutionary mode of B-type genes, D-type genes underwent more frequent gene duplications and pseudogenization (Table 1).

**Figure 6:**
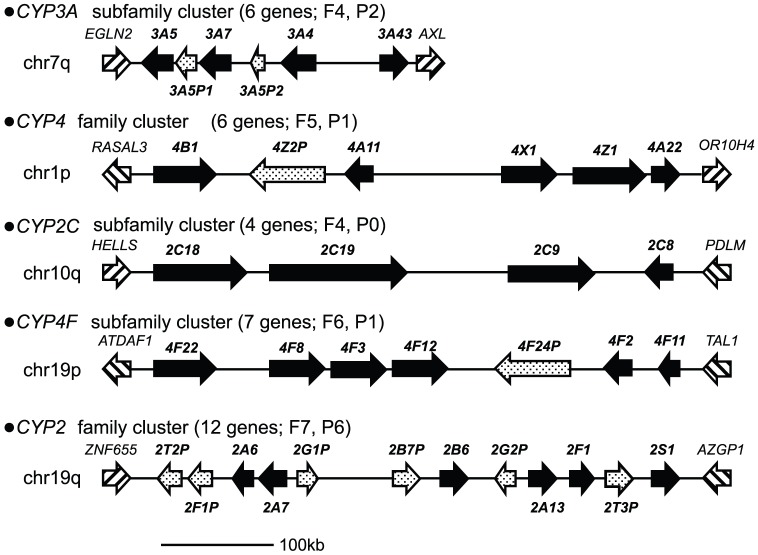
*CYP* gene clusters in the human genome. A striped arrow represents an anchor gene in a syntenic region of each cluster. Black arrows and dotted arrows represent functional *CYP* genes and pseudogenes, respectively. The length of each gene cluster is approximately 500 kb, except the *CYP3A* cluster (250 kb). The number of total genes, functional genes, and pseudogenes in each cluster are shown after the cluster name.

One important difference between D- and B-type genes is that D-type genes cluster on chromosomes, and these clusters are composed of closely related genes. This difference is reflected in the phylogeny, which shows that the genes in each cluster are monophyletic (Figure 1). In the human genome, five clusters have been identified: the *CYP2* family clusters on chromosome 19q [18], [19], the *CYP2C* subfamily clusters on chromosome 10q, the *CYP3A* subfamily clusters on chromosome 7q, the *CYP4* family clusters on chromosome 1p, and the *CYP4F* subfamily clusters on chromosome 19p. Each cluster region occupies approximately 500 kb, with the exception of *CYP3A*, which occupies 250 kb. Each cluster included the following number of genes: 12 for *CYP2*, four for *CYP2C*, six for *CYP3A* and *CYP4*; and seven for *CYP4F* (Figure 6).

**Figure 7:**
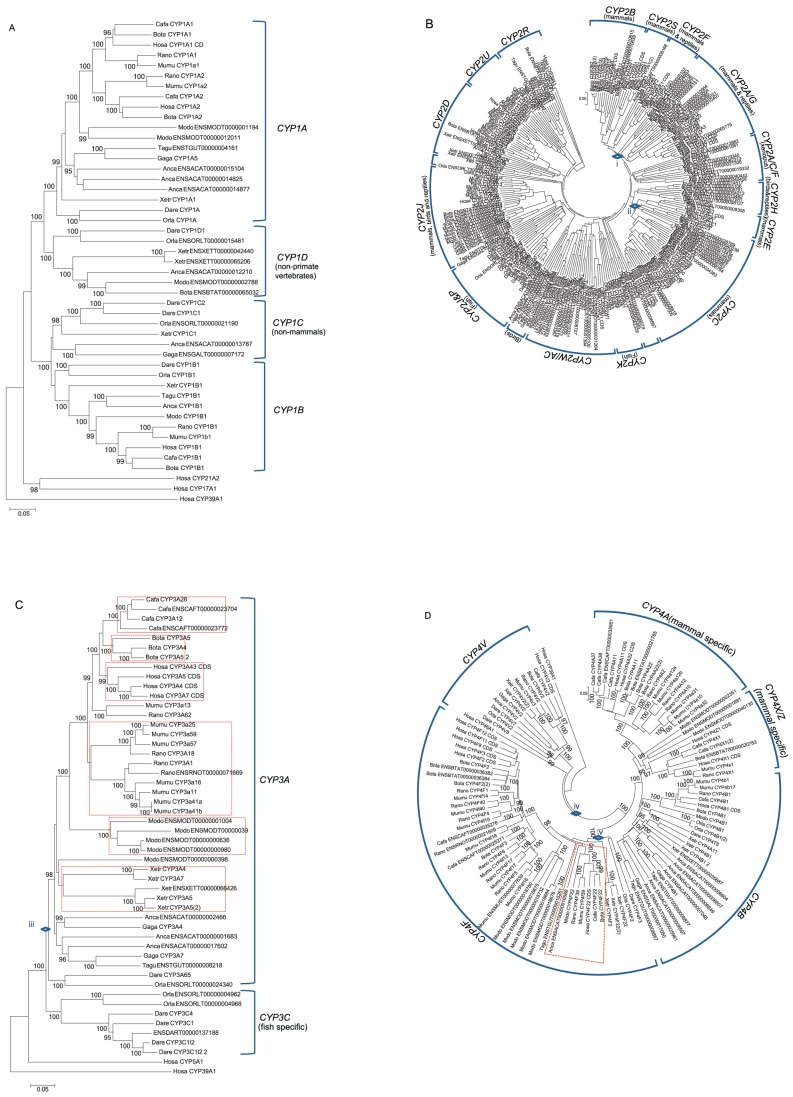
Phylogenetic tree of the D-type family. A) *CYP1* family, B) *CYP2* family, C) *CYP3* family, and D) *CYP4* family. Each NJ tree was based on the total nucleotide substitutions among members. The origin of each of the five clusters (corresponding to i–v in Figure 1) is indicated with a diamond in Figure B–D. Each subfamily is indicated by a bracket. In Figure B, the *CYP2T* subfamily is not shown because no functional gene belonging to this subfamily is present in the human genome. In Figures C and D, the red dashed rectangle outlines a specific clade.

Using the phylogenic analyses of each *CYP1–4* family in vertebrates, we identified several species-specific gene duplications. The topology of the tree for the *CYP1* family revealed four subfamilies (*1A*, *1B*, *1C*, and *1D*), and showed that these subfamilies diverged in the ancestor of vertebrates. The *CYP1A* and *1B* subfamilies were conserved from fish to humans, whereas primates lacked *CYP1D*, and mammals lacked *CYP1C* (Figure 7A). The *CYP2* family was shown to be composed of 16 subfamilies (*CYP2A*, *2B*, *2C*, *2D*, *2E*, *2F*, *2G*, *2H*, *2J*, *2K*, *2R*, *2S*, *2T*, *2U*, *2W*, and *2AC*), three of which (*CYP2B*, *2E* and *2S*) were specific to mammals, while the *2A*/*G* and *F* subfamilies were present only in mammals and reptiles. These five subfamilies (except the *CYP2E* subfamily) diverged successively to form the *CYP2* cluster in an ancestor of mammals (Figure 7B). However, *CYP2U* and *2R* were shown to be common to all vertebrates. The *CYP3* family tree contained only two subfamilies, *CYP3A* and the fish-specific *3C* family (Figure 7C). *CYP3A* comprised amphibian-, bird-, and mammal-specific clades. In each taxonomic group, members of the *CYP3A* subfamily appear to have been duplicated independently. The tree constructed for the *CYP4* family included six subfamilies (*4A*, *4B*, *4F*, *4V*, *4X*, and *4Z*) (Figure 7D). *CYP4A* and *4X/Z* were specific to mammals, whereas the other three subfamilies (*4B*, *F*, and *V*) were common to all vertebrates. In particular, the members of the *4F* subfamily formed several species-specific clusters, except *CYP4F22*. It is unclear, however, whether these species-specific clusters resulted from gene conversion or from recent duplication of the subfamily in each species. The evolution of D-type genes has involved frequent species-specific gene duplications, compared to B-type genes (Figure 5B). In D-type genes, it is unclear how many gene duplications occurred before eutherian divergence. We estimated the rate of duplication subsequent to the eutherian radiation, which revealed 53 duplications in 432 myr, or the rate of 12.7 duplications per 100 myr. No gene loss was observed. These results are in contrast to the results for B-type genes.

### B- and D-type *CYP* pseudogenes

**Figure 8:**
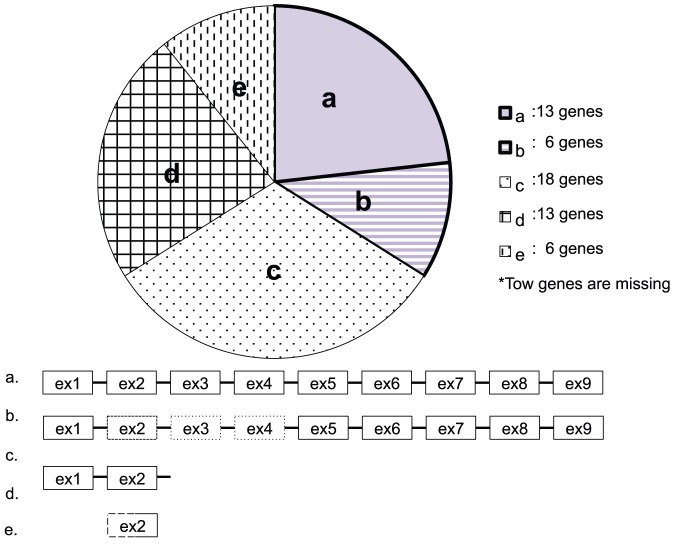
Categorization of the 58 human *CYP* pseudogenes. Among the 58 pseudogenes, paralogs were detected by a BLAST search. a. The number of exons and introns is the same as in the paralogous genes (13 genes), b. They contain greater than half the number of exons and introns of their paralogs (6 genes), c. One or two exons or introns remained (18 genes), d. A portion of an exon remained (13 genes), e. The BLAST search returned no hits (6 genes). *Two pseudogenes were absent from the human genome databases. Approximately one-third (a and b) of the human *CYP* pseudogenes were used for phylogenetic analysis.

**Figure 9:**
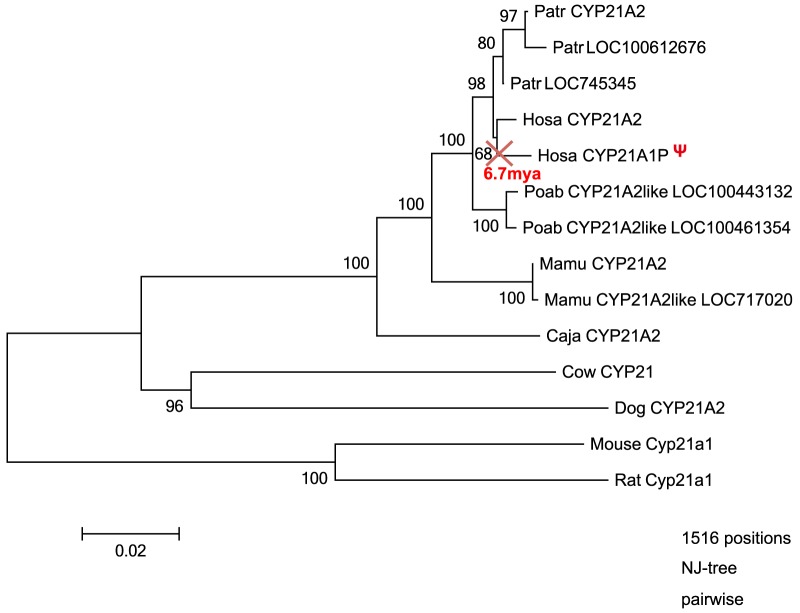
Time of pseudogenization of *CYP21A1P* in humans. The phylogenetic tree was obtained using the NJ method, using the CDS. The pseudogene is represented by Ψ. The cross shows the time at which function was lost.

The evolutionary modes of D- and B-type *CYP* genes differed also in pseudogenization, which is defined as a loss of gene function. Among the 58 pseudogenes present in the human genome, more than half (41 of 58) are fragmented, with few exons and introns remaining. The total length of such pseudogenes is less than one-tenth of that of a functional *CYP* gene, which prevented identification of several of the original genes (Figure 8). We identified the original functional genes for 17 pseudogenes, among which 3 were B-type (*CYP21A1P*, *CYP51P1*, and *CYP51P2*) and 14 were D-type (*CYP1D1P*, *CYP2A7P1*, *CYP2B7P1*, *CYP2D7P1*, *CYP2D8P1*, *CYP2F1P*, *CYP2G1P*, *CYP2G2P*, *CYP2T2P*, *CYP2T3P*, *CYP4F9P*, *CYP4F23P*, *CYP4F24P*, and *CYP4Z2P*) (Table S1). Of the 3 B-type pseudogenes, *CYP51P1* and *CYP51P2* are processed pseudogenes, and the biological causes of their pseudogenization are not related to a relaxation of functional constraints. In this sense, *CYP21A1P* is only a pseudogene due to relaxation of functional constrains. Rhesus macaques, orangutans, and humans have two copies of *CYP21A*, and chimpanzees have three (Figure 9). However, a pseudogene for *CYP21A* is present only in humans, and the time of pseudogenization was estimated to be 6.7 mya, around the divergence of humans from chimpanzees. The presence of this pseudogene is clinically significant in humans: partial gene conversion from a pseudogene to a functional gene causes 21-hydroxylase deficiency; furthermore, copy number variation has been observed in the region containing *CYP21A* and the neighboring *C4A* in the *HLA* region of human chromosome 6 [35].

In contrast, among the 14 D-type pseudogenes, four (*CYP2G1P*, *2G2P*, *2T2P*, and *2T3P*) have been reported to be human-specific, on the basis of a comparison between humans and mice [36]. We searched for orthologs to the human pseudogenes in other primate genomes and found that all but *CYP2G2P* are pseudogenized in other primates as well, but are functional in non-primate vertebrates (Figure S6). Our findings showed that *CYP2G1P*, *2T2P*, and *2T3P* are primate-specific pseudogenes, whereas *CYP2G2P* is a human-specific pseudogene. Using an accelerated non-synonymous substitution rate in pseudogenes [27], we calculated that *CYP2G2P* emerged 2.6 mya. In addition to *CYP2G2P*, further analysis revealed a single human-specific pseudogene, *4Z2P*, with a pseudogenization time of 6.4 mya. On the basis of the results of our analysis, *CYP2D7P1* also appeared to be a human-specific pseudogene. Interestingly, however, pseudogenization of this ortholog has also been found in orangutans, but the cause is different from that for humans [37]. It appears that this gene lost its function in humans and orangutans independently. In D-type genes, in addition to the 14 pseudogenes present in the human genome, 7 pseudogenes were identified in chimpanzees, macaques, marmosets, dogs, and cows. Among the seven, six were specie-specific, one (*2C18*) to chimpanzees, two (*2A13* and *4F11*) to macaques, and three (*4B1* and two *4F22*-like genes) to marmosets. The remaining *CYP2B6P* was pseudogenized independently in chimpanzees and macaques. Among the 11 pseudogenes, with the exception of the three human-specific pseudogenes, *CYP2A7P1* was pseudogenized in macaques and humans independently, at 28.4 mya and 5.9 mya, respectively. Pseudogenization of the remaining 10 genes occurred in the primate or hominoid stem lineage.

It is unclear how many times pseudogenization occurred in D-type genes before eutherian divergence. We estimated the rate subsequent to the eutherian radiation at 30 pseudogenizations over 432 myr, yielding a rate of 6.9 per 100 myr. In contrast, the number of pseudogenization events in B-type genes was estimated to be only five over 2,685 myr, yielding a rate of 0.19 per 100 myr.

### Evolutionary rate of B- and D-type genes

**Figure 10:**
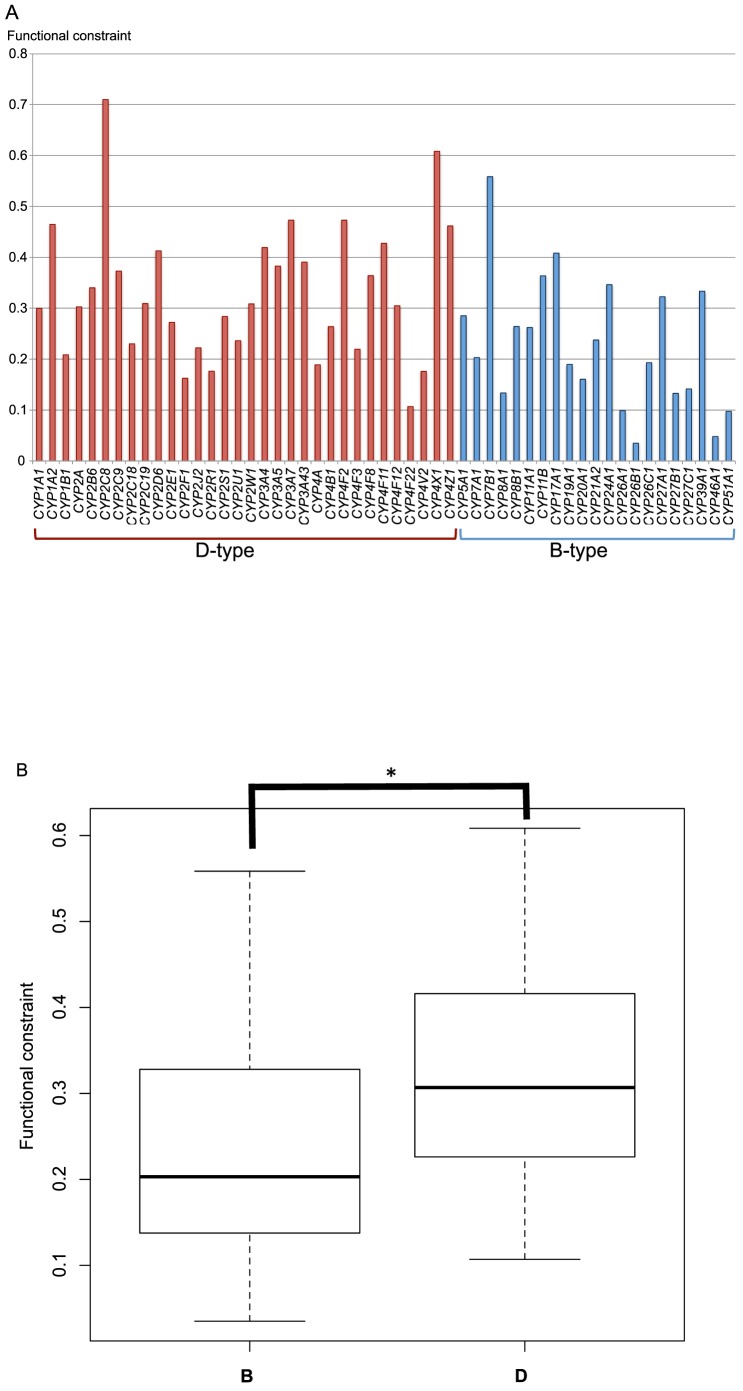
Functional constraint of *CYP* genes. A) Functional constraint was estimated for each *CYP* gene. The *y*-axis shows the functional constraint obtained via the ratio of per-site non-synonymous substitutions to synonymous substitutions (D_N_/D_S_). Red bars indicate D-type genes, and blue bars indicate B-type genes. B) Comparison of median values for functional constraints between primate B- and D-type genes. “B” indicates functional constraint of B-type genes, and “D” indicates functional constraint of D-type genes. The *P*-value was 0.01282 (significance was defined as *P*<0.05). The *P*-value was calculated using the Mann–Whitney *U* test.

Our results revealed that births and deaths of genes were more frequent in D-type genes than in B-type genes. As such, it was important to compare the evolutionary rate of B- and D-type genes. For this comparison, the non-synonymous substitution rate was normalized to the synonymous rate, and the ratio (*f*) for each B- and D-type gene was calculated in primates (see Materials and Method, Figure 10A). The average *f*-values for B- and D-type genes were calculated to be 0.24±0.14 and 0.33±0.13, respectively, and the median values for B- and D-type genes were determined to be 0.23 and 0.31, respectively (Figure 10B). The average and median values for D-type genes were significantly greater than those for B-type genes (Wilcoxon's test, *P*-value = 0.0173), suggesting that the degree of functional constraint (1-*f*) is stronger in B-type than in D-type genes. These results are consistent with the rapid birth and death process of D-type genes.

## Discussion

### The origin of D-type *CYP* genes

The origin of B-type genes is assumed to be single and ancient, because fission yeast possesses B-type genes and because a possible ortholog to the B-type gene *CYP51* is present even in prokaryotic genomes. However, D-type genes are thought to have different origins. The present phylogenetic analysis demonstrated that four D-type families are conserved among all vertebrates, and that the D-type families were derived from three gene-duplication events of B-type genes in the stem lineage of vertebrates. Based on the molecular clock hypothesis, B- to D-type gene duplications occurred approximately 600–700 mya, consistent with the phylogenetic analysis. However, the D-type *CYPs* impart resistance to insecticides in invertebrates; in fruit flies, two such enzymes are CYP6U1 and CYP6D2. The phylogenetic analysis of both human and fruit fly *CYP* genes indicated an independent emergence of D-type genes. Moreover, other invertebrate genomes contain human D-type-like genes, but orthology has not been confirmed. It appears that D-type genes in vertebrates and insects evolved independently from different origins, which is consistent with the idea of a rapid turnover of D-type genes.

In this paper, we focused on an early stage of *CYP* gene diversification in vertebrates and showed the emergence of D-type from B-type genes. However, some exceptions should be noted. For example, *CYP2R1* is categorized as a B-type gene on the basis of its function, but the nucleotide sequence showed that it is closely related to other D-type *CYP2* genes. From this observation, it appears that *CYP2R1* has been converted from a D-type to a B-type *CYP* gene. This is supported by the observation that the amino acid sequence of CYP2R1 is highly conserved in all vertebrates, reflecting the high degree of functional constraint against the gene.

### The evolution of *CYP* genes is driven by substrate specificity

The birth and death (pseudogenization) rates of B- and D-type genes differed in magnitude: the rates in B-type genes were 0.7 and 0.2 per 100 myr, respectively, whereas those in D-type genes were 12.7 and 6.9 per 100 myr, respectively. Compared with D-type genes, the evolution of B-type genes was highly conserved with regard to their mode of birth and death processes as well as amino acid substitutions. The substrates of B-type enzymes are chemicals that play important roles in metabolism of vitamin D, steroids, and cholesterol. In contrast, the substrates of D-type enzymes are xenobiotics such as plant alkaloids. In light of this substrate specificity, we hypothesize that the conserved evolutionary pattern observed in B-type enzymes reflects the importance and conservation of their substrates, whereas the rapid evolution of D-type enzymes indicates that their substrates are flexible and highly dependent on environmental factors. Future studies of the evolution of the substrate-recognition sites will be required to confirm this hypothesis.

## Supporting Information

Figure S1 **Conserved amino acids at a position.** The *x*-axis indicates amino-acid positions in an alignment of 710 vertebrate *CYP* genes (after excluding gaps), and the y-axis indicates the ratio of conserved amino acids at each position. Red bars indicate highly (>95%) conserved positions. The chart below the bar graph reports the approximate position of six substrate recognition sites (SRS): SRS1–6. The bracket represents the heme-binding region (∼10 amino acids) of the *CYP* gene.
(EPS)

Figure S2 **Conserved amino acids within each clan of vertebrate CYP genes.** Conserved amino acids within each clan are shown in colored columns: the red column is specific for clan 2; aqua, mito; orange, 4; blue, 3; yellow, 26, and brown, 7. The four sites in purple (E, R, G, and C) are highly conserved (>95%) sites among all vertebrate species. This figure shows only human *CYP* genes: positions are 275–285 and 350–360.
(EPS)

Figure S3 **Amino acids distinguishing B-type from D-type genes within each clan.** The yellow column indicates the amino acids in B-type genes that differ from D-type genes. The other colors are the same as in Figure S2. A, Clan 2; B, Clan 3; C, Clan 4 and 46.
(ZIP)

Figure S4 **Conserved amino acids between human and** ***Drosophila*** **genes in a** ***CYP19*** **clan.** Human (*CYP19A1*) and Drosophila (*CYP313A1*, *313B1*, *316A1*, and *318A1*) genes share the same amino acids at 16 of 423 aligned sites. The shared sites are shown in red.
(EPS)

Figure S5 **Phylogenetic tree and species-divergence time.** The phylogenetic tree was constructed on the basis of the divergence time of 15 species (time tree). The species names at the tip of the tree are abbreviated as in Table 1. *Hosa*, *Homo sapiens*. The number at each node represents species divergence time in mya. The scale under the tree indicates time in myr.
(EPS)

Figure S6 **The cause of functional loss in human-specific** ***CYP*** **pseudogenes.** There are four human-specific *CYP* psuedogenes (*CYP2G1P*, *2P*, *CYP2T2P*, and *3P*). Possible causal mutations, premature stop codons (red) and frame-shift mutations (blue), were identified in human and other primate CYP nucleotide and amino acid alignments. The row labeled “exon” for *CYP2G1P* and *2P* shows the number of exons in which mutations were found, and the row labeled “bp” indicates the nucleotide position of the CDS in functional genes from rat and mouse.
(EPS)

Table S1 **The number of** ***CYP*** **gene in Human.** After exclusion of truncated pseudogeens each category includes genes as below. **a**: *CYP1A1*, *CYP1A2*, *CYP1B1*, *CYP2A6*, *CYP2A7*, *CYP2B6*, *CYP2C8*, *CYP2C9*, *CYP2C18*, *CYP2C19*, *CYP2D6*, *CYP2E1*, *CYP2F1*, *CYP2J2*, *CYP2R1*, *CYP2S1*, *CYP2U1*, *CYP2W1*, *CYP3A4*, *CYP3A5*, *CYP3A7*, *CYP3A43*, *CYP4A11*, *CYP4A20*, *CYP4A22*, *CYP4B1*, *CYP4F2*, *CYP4F3*, *CYP4F8*, *CYP4F11*, *CYP4F12*, *CYP4F22*, *CYP4V2*, *CYP4X1*, **b**: *CYP1D1P*, *CYP2A7P1*, *CYP2B7P1*, *CYP2D7P1*, *CYP2D8P1*, *CYP2F1P*, *CYP2G1P*, *CYP2G2P*, *CYP2T2P*, *CYP2T3P*, *CYP4F9P*, *CYP4F23P*, *CYP4F24P*, *CYP4Z2P*, **c**: *CYP5A1*, *CYP7A1*, *CYP7B1*, *CYP8A1*, *CYP8B1*, *CYP11A1*, *CYP11B1*, *CYP11B2*, *CYP17A1*, *CYP19A1*, *CYP20A1*, *CYP21A2*, *CYP24A1*, *CYP26A1*, *CYP26B1*, *CYP26C1*, *CYP27A1*, *CYP27B1*, *CYP27C1*, *CYP39A1*, *CYP46A1*, *CYP51A1*, **d**: *CYP21A1P*, *CYP51P1*, *CYP51P2*.
(DOCX)

File S1 **The list of human CYP gene names and their accession numbers in this article.**
(DOCX)

## References

[1] Klingenberg M (1958) Pigments of rat liver microsomes. Arch Biochem Biophys 75: 376–386. 13534720 10.1016/0003-9861(58)90436-3

[2] Omura T, Sato R (1962) A new cytochrome in liver microsomes. J Biol Chem 237: PC1375–PC1376. 14482007

[3] Nelson DR (1998) Metazoan cytochrome P450 evolution. Comp Biochemi and Phys Part C: Pharm Toxic Endocr 121: 15–22. 10.1016/s0742-8413(98)10027-0 9972448

[4] Gotoh O (2012) Evolution of cytochrome p450 genes from the viewpoint of genome informatics. Biol Pharm Bull 35: 812–817. 22687468 10.1248/bpb.35.812

[5] Munro AW, Lindsay G (1996) Bacterial cytochromes P-450. Mol Microbiol 20: 1115–1125. 8809764 10.1111/j.1365-2958.1996.tb02632.x

[6] Nelson DR (2009) The cytochrome p450 homepage. Human Genomics 1: 59–65. 10.1186/1479-7364-4-1-59 PMC3500189 19951895

[7] Sea Urchin Genome Sequencing Consortium (2006) The genome of the sea urchin *Strongylocentrotus purpuratus* . Science 314: 941–952. 17095691 10.1126/science.1133609 PMC3159423

[8] Mao G, Seebeck T, Schrenker D, Yu O (2013) CYP709B3, a cytochrome P450 monooxygenase gene involved in salt tolerance in *Arabidopsis thaliana* . BMC Plant Biol 13: 169. 24164720 10.1186/1471-2229-13-169 PMC3819737

[9] Nelson DR, Goldstone JV, Stegeman JJ (2013) The cytochrome P450 genesis locus: the origin and evolution of animal cytochrome P450s. Phil Trans R Soc B 368: 20120474. 23297357 10.1098/rstb.2012.0474 PMC3538424

[10] Quaderer R, Omura S, Ikeda H, Cane DE (2006) Pentalenolactone biosynthesis. Molecular cloning and assignment of biochemical function to PtlI, a cytochrome P450 of Streptomyces avermitilis. J Am Chem Soc 128: 13036–13037. 17017767 10.1021/ja0639214 PMC2533730

[11] Aoyama Y, Horiuchi T, Gotoh O, Noshiro M, Yoshida Y (1998) CYP51-like gene of *Mycobacterium tuberculosis* actually encodes a P450 similar to eukaryotic CYP51. J Biochem 124: 694–696. 9756611 10.1093/oxfordjournals.jbchem.a022167

[12] Yoshida Y, Aoyama Y, Noshiro M, Gotoh O (2000) Sterol 14-demethylase P450 (CYP51) provides a breakthrough for the discussion on the evolution of cytochrome P450 gene superfamily. Biochem Biophys Res Comm 273: 799–804. 10891326 10.1006/bbrc.2000.3030

[13] Debeljak N, Fink M, Rozman D (2003) Many facets of mammalian lanosterol 14α-demethylase from the evolutionarily conserved cytochrome P450 family CYP51. Arch Biochem Biophys 409: 159–171. 12464255 10.1016/s0003-9861(02)00418-6

[14] Qi X, Bakht S, Qin B, Leggett M, Hemmings A, et al (2006) A different function for a member of an ancient and highly conserved cytochrome P450 family: From essential sterols to plant defense. Proc Natl Acd Sci USA 103: 18848–18853. 10.1073/pnas.0607849103 PMC1656972 17124172

[15] Nelson DR (1999) Cytochrome P450 and the individuality of species. Arch Biochem Biophys 369: 1–10. 10462435 10.1006/abbi.1999.1352

[16] Rezen T, Debeljak N, Kordis D, Rozman D (2004) New aspects of lanosterol 14a-demethlase and cytochrome P450 evolution: Lanosterol/cycloartenol diversification and lateral transfer. J Mol Evol 59: 51–58. 15383907 10.1007/s00239-004-2603-1

[17] Nebert DW, Dalton TP (2006) The role of cytochrome P450 enzymes in endogenous signaling pathways and environmental carcinogenesis. Nature Rev Cancer 6: 947–960. 17128211 10.1038/nrc2015

[18] Hoffman SMG, Hu S (2006) Dynamic evolution of the CYP2ABFGST gene cluster in primates. Mutation Res 616: 133–138. 17194463 10.1016/j.mrfmmm.2006.11.004

[19] Hu S, Wang H, Knisely AA, Raddy S, Kovacevic D, et al (2008) Evolution of the CYP2ABFGST gene cluster in rat, and a fine-scale comparison among rodent and primate species. Genetica 133: 215–226. 17876710 10.1007/s10709-007-9206-x

[20] Thomas JH (2007) Rapid birth-death evolution specific to xenobiotic cytochrome P450 genes in vertebrates. PLoS Genetics 3: e67. 17500592 10.1371/journal.pgen.0030067 PMC1866355

[21] Larkin MA, Blackshields G, Brown NP, Chenna R, McGettigan PA, et al (2007) Clustal W and Clustal X version 2.0. Bioinformatics 23: 2947–2948. 17846036 10.1093/bioinformatics/btm404

[22] Tamura K, Peterson D, Peterson N, Stecher G, Nei M, et al (2011) MEGA5: Molecular evolutionary genetics analysis using maximum likelihood, evolutionary distance, and maximum parsimony methods. Mol Biol Evol 28: 2731–2739. 21546353 10.1093/molbev/msr121 PMC3203626

[23] Saitou N, Nei M (1987) The neighbor-joining method: a new method for reconstructing phylogenetic trees. Mol Biol Evol 4: 406–425. 3447015 10.1093/oxfordjournals.molbev.a040454

[24] Nei M and Kumar S (2000). Molecular Evolution and Phylogenetics. Oxford University Press, New York.

[25] Jones D, Taylor WR, Thronton JM (1992) The rapid generation of mutation data matrices from protein sequences. Comput Appl Biosci 8: 275–282. 1633570 10.1093/bioinformatics/8.3.275

[26] Felsenstein J (1981) Evolutionary trees from DNA sequences: a maximum likelihood approach. J Mol Evol 17: 368–76. 7288891 10.1007/BF01734359

[27] Sawai H, Go Y, Satta Y (2008) Biological implication for loss of function at major histocompatibility complex loci. Immunogenetics 60: 295–302. 18461313 10.1007/s00251-008-0291-5

[28] Hedges SB, Dudley, Kumar S (2006) Time Tree: a public knowledge-base of divergence times among organisms. Bioinformatics 23: 2971–2. 10.1093/bioinformatics/btl505 17021158

[29] Rzhetsky A, Nei M (1992) Statistical properties of the ordinary least-squares, generalized least-squares, and minimum-evolution methods of phylogenetic inference. J Mol Evol 35: 367–75. 1404422 10.1007/BF00161174

[30] Mann HB, Whitney DR (1947) On a test of whether one of two random variables is stochastically larger than the other. Ann Math Statist 18: 50–60.

[31] Ohmura T, Ishimura Y, Fujii Y (2009) Molecular biology of P450. 2^nd^ ed.

[32] Nelson DR, Koymans L, Kamataki T, Stegeman JJ, Feyereisen R, et al (1996) P450 superfamily: update on new sequences, gene mapping, accession numbers and nomenclature. Pharmacogenetics 6 1: 1–42. 8845856 10.1097/00008571-199602000-00002

[33] Meunier B, de Visser SP, Shaik S (2004) Mechanism of oxidation reactions catalyzed by cytochrome P450 enzymes. Chem Rev 104: 3947–3980. 15352783 10.1021/cr020443g

[34] Feyereisen R (2011) Arthropod CYPomes illustrate the tempo and mode in P450 evolution. Bioch et Biophys Acta 1814: 19–28. 10.1016/j.bbapap.2010.06.012 20601227

[35] Urabe K, Kimura A, Harada F, Iwanaga T, Sasazuki T (1990) Gene conversion in steroid 21-hydroxylase genes. Am J Hum Genet 46: 1178–1186. 1971153 PMC1683832

[36] Nelson DR, Zeldin DC, Hoffman SM, Maltais LJ, Wain HM, et al (2004) Comparison of cytochrome P450 (CYP) genes from the mouse and human genomes, including nomenclature recommendations for genes, pseudogenes and alternative-splice variants. Pharmacogenetics 14 1: 1–18. 15128046 10.1097/00008571-200401000-00001

[37] Yasukochi Yoshiki, Satta Yoko (2011) Evolution of the CYP2D gene cluster in humans and four non-human primates. Genes Genet Syst 86: 109–116. 21670550 10.1266/ggs.86.109

