# Supplementary material for: Substrate-Dependent Evolution of Cytochrome P450: Rapid Turnover of the Detoxification-Type and Conservation of the Biosynthesis-Type
Source: PLoS One. 2014 Jun 30;9(6):e100059. doi: 10.1371/journal.pone.0100059 (PMC4076195; doi:10.1371/journal.pone.0100059)
Supplement: Table S1 — The number of CYP gene in Human. After exclusion of truncated pseudogeens each category includes genes as below. a: CYP1A1, CYP1A2, CYP1B1, CYP2A6, CYP2A7, CYP2B6, CYP2C8, CYP2C9, CYP2C18, CYP2C19, CYP2D6, CYP2E1, CYP2F1, CYP2J2, CYP2R1, CYP2S1, CYP2U1, CYP2W1, CYP3A4, CYP3A5, CYP3A7, CYP3A43, CYP4A11, CYP4A20, CYP4A22, CYP4B1, CYP4F2, CYP4F3, CYP4F8, CYP4F11, CYP4F12, CYP4F22, CYP4V2, CYP4X1, b: CYP1D1P, CYP2A7P1, CYP2B7P1, CYP2D7P1, CYP2D8P1, CYP2F1P, CYP2G1P, CYP2G2P, CYP2T2P, CYP2T3P, CYP4F9P, CYP4F23P, CYP4F24P, CYP4Z2P, c: CYP5A1, CYP7A1, CYP7B1, CYP8A1, CYP8B1, CYP11A1, CYP11B1, CYP11B2, CYP17A1, CYP19A1, CYP20A1, CYP21A2, CYP24A1, CYP26A1, CYP26B1, CYP26C1, CYP27A1, CYP27B1, CYP27C1, CYP39A1, CYP46A1, CYP51A1, d: CYP21A1P, CYP51P1, CYP51P2. (DOCX) [file pone.0100059.s007.docx]

**Table S1. The number of *CYP* gene in Human**

|  | Functional gene | Pseudogene |
| --- | --- | --- |
| Detoxification | 35^a^ | 14^b^ |
| Biosynthesis | 22^c^ | 3^d^ |
