# Supplementary material for: Substrate-Dependent Evolution of Cytochrome P450: Rapid Turnover of the Detoxification-Type and Conservation of the Biosynthesis-Type
Source: PLoS One. 2014 Jun 30;9(6):e100059. doi: 10.1371/journal.pone.0100059 (PMC4076195; doi:10.1371/journal.pone.0100059)
Supplement: File S1 — The list of human CYP gene names and their accession numbers in this article. (DOCX) [file pone.0100059.s008.docx]

**Supplemental information 1.** **The list of human CYP gene names and their accession numbers in this article**

*CYP1A1* (NM_000499)*, CYP1A2* (NM_000761)*, CYP1B1* (NM_000104)*, CYP2A6* (NM_000762)*, CYP2A7* (NM_000764)*, CYP2B6* (NM_000767)*, CYP2C8* (NM_000770)*, CYP2C9* (NM_000771)*, CYP2C18* (NM_000772)*, CYP2C19* (NM_000769)*, CYP2D6* (NM_000106)*, CYP2E1* (NM_000773)*, CYP2F1* (NM_000774)*, CYP2J2* (NM_775)*, CYP2R1* (NM_024514)*, CYP2S1* (NM_030622)*, CYP2U1* (NM_183075)*, CYP2W1* (NM_017781)*, CYP3A4* (NM_001202855)*, CYP3A5* (NM_000777)*, CYP3A7* (NM_000765)*, CYP3A43* (NM_001278921)*, CYP4A11* (NM_000778)*, CYP4A20* (NM_178134)*, CYP4A22* (NM_001010969)*, CYP4B1* (NM_000779)*, CYP4F2* (NM_001082)*, CYP4F3* (NM_000896)*, CYP4F8* (NM_007253)*, CYP4F11* (NM_001128932)*, CYP4F12* (NM_023944)*, CYP4F22* (NM_173483)*, CYP4V2* (NM_203752)*, CYP4X1* (NM_178033)*, CYP5A1* (NM_001061)*, CYP7A1* (NM_000780)*, CYP7B1* (NM_004820)*, CYP8A1* (NM_000961)*, CYP8B1* (NM_004391)*, CYP11A1* (NM_000781)*, CYP11B1* (NM_000497)*, CYP11B2* (NM_000498)*, CYP17A1* (NM_000102)*, CYP19A1* (NM_000103)*, CYP20A1* (NM_177538)*, CYP21A2* (NM_000500)*, CYP24A1* (NM_000782)*, CYP26A1* (NM_000783)*, CYP26B1* (NM_001277742)*, CYP26C1* (NM_183374)*, CYP27A1* (NM_000784)*, CYP27B1* (NM_000785)*, CYP27C1* (NM_001001665)*, CYP39A1* (NM_001278738)*, CYP46A1* (NM_006668)*, CYP51A1* (NM_000786)
